# Effect of single and mixed polycyclic aromatic hydrocarbon contamination on plant biomass yield and PAH dissipation during phytoremediation

**DOI:** 10.1007/s11356-018-1987-1

**Published:** 2018-04-27

**Authors:** Seniyat Larai Afegbua, Lesley Claire Batty

**Affiliations:** School of Geography, Earth and Environmental Sciences, College of Life and Environmental Sciences, University of Birmingham, Birmingham, B15 2TT UK

**Keywords:** Phytoremediation, Phenanthrene, Fluoranthene, Benzo[*a*]pyrene, *Medicago sativa*, *Lolium perenne*, *Festuca arundinacea*, Inhibition

## Abstract

Polycyclic aromatic hydrocarbon (PAH)-contaminated sites have a mixture of PAH of varying concentration which may affect PAH dissipation differently to contamination with a single PAH. In this study, pot experiments investigated the impact of PAH contamination on *Medicago sativa*, *Lolium perenne*, and *Festuca arundinacea* biomass and PAH dissipation from soils spiked with phenanthrene (Phe), fluoranthene (Flu), and benzo[*a*]pyrene (B[*a*]P) in single and mixed treatments. Stimulatory or inhibitory effects of PAH contamination on plant biomass yields were not different for the single and mixed PAH treatments. Results showed significant effect of PAH treatments on plant growth with an increased root biomass yield for *F. arundinacea* in the Phe (175%) and Flu (86%) treatments and a root biomass decrease in the mixed treatment (4%). The mean residual PAHs in the planted treatments and unplanted control for the single treatments were not significantly different. B[*a*]P dissipation was enhanced for single and mixed treatments (71–72%) with *F. arundinacea* compared to the unplanted control (24–50%). On the other hand, B[*a*]P dissipation was inhibited with *L. perenne* (6%) in the single treatment and *M. sativa* (11%) and *L. perenne* (29%) in the mixed treatment. Abiotic processes had greater contribution to PAH dissipation compared to rhizodegradation in both treatments. In most cases, a stimulatory effect of PAH contamination on plant biomass yield without an enhancement of PAH dissipation was observed. Plant species among other factors affect the relative contribution of PAH dissipation mechanisms during phytoremediation. These factors determine the effectiveness and suitability of phytoremediation as a remedial strategy for PAH-contaminated sites. Further studies on impact of PAH contamination, plant selection, and rhizosphere activities on soil microbial community structure and remediation outcome are required.

## Introduction

Recently, there has been a marked increase in research on phytoremediation as a promising eco-friendly remediation technology. This has been driven by reports of enhanced biodegradation of organic compounds including PAH in the presence of plants compared to unplanted soils (Siciliano et al. 2003; Xu et al. 2006; Olson et al. 2007; Vangronsveld et al. 2009; Wu et al. 2011). An enhanced dissipation in vegetated soils is attributed to rhizospheric effect through root exudation providing benefit such as improved soil condition, bioavailability, and stimulation of microbial activity (Kirk et al. 2005; Kaimi et al. 2006; Cheema et al. 2010; Hamdi et al. 2012). Apart from microbial degradation and rhizodegradation, abiotic processes such as volatilization, leaching, and adsorption to soil fractions may contribute to PAH loss (Kaimi et al. 2006).

PAH concentration in contaminated soils and plant tolerance level may influence plant biomass yield and PAH degradation as a result of the impact on seed germination, plant establishment, and growth (Smith et al. 2006; Lee et al. 2008; Gan et al. 2009). Interestingly, there are conflicting reports on the phytoremediation outcome (enhancement or inhibition) as a few studies have shown that presence of plants may not necessarily enhance PAH dissipation (Sun et al. 2010; Smith et al. 2011). Further, there are few studies on the contribution of different dissipation mechanisms during phytoremediation (Sun et al. 2010; Smith et al. 2011). Many phytoremediation studies have shifted towards mixed contamination remediation to reflect real site remediation scenarios as early studies were mainly on single contaminant remediation (Gan et al. 2009).

The aim of this study was to assess the impact of single and mixed PAH treatments on plant biomass and PAH dissipation and the contribution of abiotic processes and rhizodegradation to PAH dissipation during a greenhouse experiment. Soils were spiked with phenanthrene, fluoranthene, and benzo[*a*]pyrene in single and mixed treatments. *Medicago sativa*, *Lolium perenne*, and *Festuca arundinacea* were selected for this study based on their rhizodegradation potential attributed to their root structure and stress tolerance level in previous studies (Kaimi et al. 2006; Cheema et al. 2010; Lu et al. 2011). The following hypotheses were made; single PAH and mixed PAH treatments will affect biomass yields and PAH dissipation for selected plants but greater impacts would be observed in the mixed PAH treatment. Following the greenhouse experiments, mean residual PAH concentration of the different treatments will differ between vegetated soils and non-vegetated soils. PAH loss would be attributed to different dissipation pathways (abiotic processes and rhizodegradation**)**.

## Materials and methods

### Chemicals

Phenanthrene (> 98% purity), fluoranthene (> 98% purity), and benzo[*a*]pyrene (> 96% purity) were obtained from VWR, UK. Internal standard mix (acenaphthene-*d10*, chrysene-*d12*, 1,4-dichlorobenzene-*d4*, naphthalene-*d8*, perylene-*d12*, and phenanthrene-*d10*), *p*-terphenyl-*d14*, and New Jersey Department of Environmental Protection (NJDEP) extractable petroleum hydrocarbon aromatics calibration standard 10/08 Rev.2 (2000 μg mL^−1^ each of acenaphthene, acenaphthylene, anthracene, benzo[*a*]anthracene, benzo[*a*]pyrene, benzo[*b*]fluoranthene, benzo[*g,h.i*]perylene, benzo[*k*]fluoranthene, chrysene, dibenzo[*a,h*]anthracene, fluoranthene, fluorene, indeno[*1,2,3-cd*]pyrene, 2-methylnaphthalene, naphthalene, phenanthrene, pyrene, and 1,2,3-trimethylbenzene in methylene chloride) of chromatographic grade were purchased from Restek, UK. Other chemicals used were of analytical purity.

### Soil preparation and experimental design

Sandy loam soil (pH 7.5, organic matter 6.19%, conductivity 1450 μS, moisture content 0.80%) sourced from a commercial supplier (Travis Perkins, UK) was air-dried and sieved (< 2 mm) prior to spiking with phenanthrene, fluoranthene, and benzo[*a*]pyrene for single and mixed PAH treatments in triplicate. The single PAH treatment was prepared by spiking 250 g of soil with phenanthrene (300 mg), fluoranthene (200 mg), and benzo[*a*]pyrene (5 mg) dissolved in 20 mL of acetone. Mixed PAH treatment used 250 g of soil spiked with all three compounds (phenanthrene, 300 mg; fluoranthene, 200 mg; benzo[*a*]pyrene, 5 mg) dissolved in 20 mL of acetone. The spiked soils were mixed and air-dried in a fume hood for 3 days before mixing with 750 g of unspiked soil and sieved through a 2-mm mesh and mixed thoroughly to achieve homogeneity (Cheema et al. 2010). The spiked soils were stored in the dark at room temperature for 4 weeks for equilibration before the pot experiment. The PAH spiked soils and control soils (without PAH) were dispensed into *Desch* plant plastic pot (diameter, 14 cm; depth, 12.4 cm; and capacity, 1.3 l) with 1 kg dry weight soil pot^−1^. This was followed by the transplantation of 4-week old seedlings of *Medicago sativa*, *Festuca arundinacea*, and *Lolium perenne* in perlite. Plants were grown in a controlled environment growth chamber for 65 days (16 h, 25 °C day: 8 h, 20 °C night). Pots were watered as required and excess water collected in saucers. Fertilizer was not applied during the experiment. Abiotic controls were set up in triplicate using unplanted spiked soil with formalin (30 mL) added weekly to inhibit microbial growth (Sun et al. 2010). Abiotic controls assessed contribution made by abiotic processes while unplanted spiked controls without formalin were also set up in triplicate to assess contribution of abiotic processes as well as microbial degradation to PAH dissipation**.** The plant seedlings were thinned after 2 weeks to 20 seedlings per pot. Soil samples were collected before and after the 65-day greenhouse experiment for initial and final PAH concentration following thorough mixing to ensure homogeneity. For the initial PAH concentration, six soil samples were collected from the spiked soils of each treatment group. For the final soil PAH concentration, plants were harvested and shaken to remove loosely adhering soils. Rhizosphere soil samples were taken and stored at 4 °C prior to analyses.

### Plant biomass

Following the harvest, plant roots were washed gently with water and then with deionized water to remove rhizosphere soil and the excess water blotted off roots with clean dry tissue paper. The plant materials were oven-dried to a constant weight at 65 °C for 48 h and weighed using a weighing balance (Mettler, UK) for biomass calculation (Chigbo et al. 2013).

### PAH analysis

#### Microwave extraction and solid phase extraction

Sodium sulfate (7 g) was added to soil samples (5 g) to remove any moisture and followed by addition of 15 mL of 2:1 hexane:acetone mixture, 5 mL of 1:4 triethylamine:acetone mixture, and *p*-terphenyl-*d14* in a microwave extraction tube (Chigbo et al. 2013). The content of the tube was mixed using a vortex mixer (VWR, UK) and shaken by inversion to dislodge soil material from the base. Extraction was carried out with a microwave extraction unit (CEM MARS) with the following conditions: temperature ramp to 100 °C at 800 W for 12 min, hold at 100 °C at 800 W for 10 min then cool for 5 min in accordance with the USEPA method 3546 (USEPA 2007). Following the extraction, the clear extracts were transferred into glass tubes (20 mL). For the solid phase extraction, SPE HF Mega BE-SI 2 g 12 mL cartridges (Agilent, UK) were conditioned with 5 mL of hexane then, 1 mL of sample extract was added and eluted with 10 mL of 1:1 hexane:dichloromethane mixture. The eluant was collected in a clean 20 mL glass tube and concentrated to a final volume of 1 mL under a gentle stream of nitrogen gas. Samples were prepared in 2 mL vials (Agilent, UK) by adding a semi-volatile internal standard mix to the concentrated sample extracts from soil samples for GC-MS analysis.

#### GC-MS analysis

PAH analysis was performed with an Agilent gas chromatograph-mass selective detector (Agilent Technologies 6890N Network GC System) with HP 5MS fused silica capillary column of dimensions 30 m × 0.25 mm i.d. × 0.25 μm film thickness (Agilent, UK). The GC-MS was operated in selective ion mode using operating conditions for USEPA method 8270D with helium as a carrier gas at a constant flow rate of 30 cm s^−1^ (USEPA 2014). PAH quantification was achieved in comparison with a standard curve for aromatics calibration standard and internal standard mixture (1, 4-dichlorobenzene-*d4*, naphthalene-*d8*, acenaphthene-*d10*, phenanthrene-*d10*, chrysene-*d12*, and perylene-*d12*) while *p*-terphenyl-*d14* was used as the surrogate standard. The calibration points were 50, 100, 500, 1000, 2000, 5000, and 10,000 pg μL^−1^. Quality controls were set up with solvent blanks and matrix spikes. Percentage recovery for surrogate standard *p*-terphenyl was 46.04–93.3%.

### Data analysis

Plant biomass and soil analyses data are presented as mean and standard error of replicate samples. Statistical analyses were carried out on the plant biomass and residual PAH concentration data using ANOVA followed by a Tukey Honest Significant Difference (HSD) post hoc test at a significance level of 0.05 on Statistical Package for Social Science (SPSS) (version 20.0 for Windows). The percentage PAH dissipation in each treatment equals total concentration of PAH dissipated divided by initial PAH concentration expressed in percentage. The proportion of overall dissipation attributable to plants and microbes (rhizodegradation) is calculated as difference between total dissipation from planted experiment and abiotic control expressed as a percentage of initial concentration.

## Results

### Plant biomass yield

**Fig. 1:**
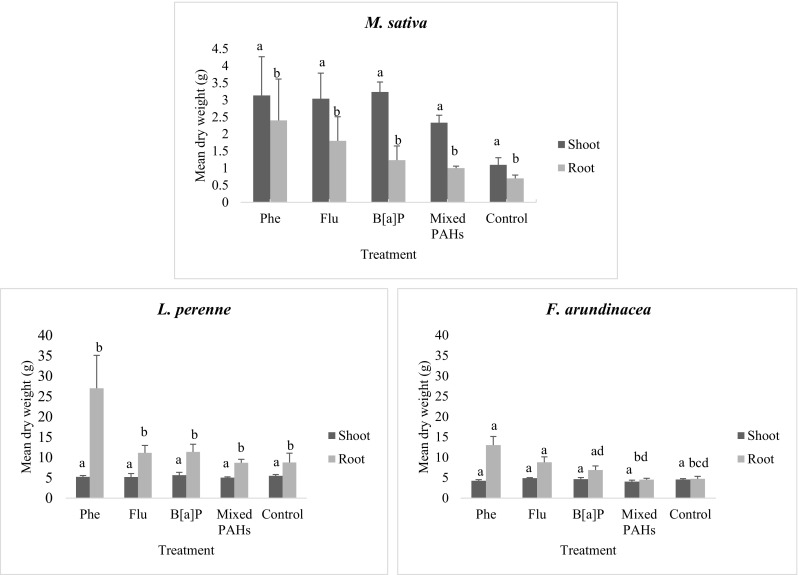
Mean shoot and root biomass of plants (g) of *M. sativa*, *L. perenne*, and *F. arundinacea* grown on soils with phenanthrene (Phe), fluoranthene (Flu), benzo[*a*]pyrene (B[*a*]P), and phenanthrene + fluoranthene + benzo[*a*]pyrene (Mixed PAH) after 65 days of growth. Error bars represent standard error of three sampled pots with 20 seedlings each. Different letters indicate a significant difference (*p* = 0.05). *M. sativa* shoot biomass *p* > 0.05 and root biomass *p* > 0.05. *L. perenne* shoot biomass *p* > 0.05 and root biomass *p* > 0.05. *F. arundinacea* shoot biomass *p* > 0.05 and root biomass *p* < 0.01

*M. sativa* shoot biomass yield increase was 190, 180, 190, and 110% for Phe, Flu, B[*a*]P, and mixed PAH treatments respectively compared to the control. *M. sativa* root biomass yield increase relative to the control was 240, 160, 80, and 40% for the Phe, Flu, B[*a*]P, and mixed PAH treatments respectively. Root biomass yield decrease was observed for *L. perenne* with 5, 6, and 8% for Phe, Flu, and mixed PAH treatments respectively compared to the control. *L. perenne* root biomass increases observed were 210, 30, and 30% for Phe, Flu, and B[*a*]P treatments respectively while a decrease (0.7%) was observed for the mixed PAH treatment. The effect of the single and mixed PAH treatments on the root and shoot biomass of *M. sativa* and *L. perenne* compared to the control plants was not significant (*p* > 0.05). One-way ANOVA showed that the effect of PAH treatments on *F. arundinacea* shoot biomass was not significant (*p* > 0.05) while that for *F. arundinacea*, root biomass (*p* < 0.01) was significant (Fig. 1). There was a decrease in *F. arundinacea* shoot biomass yield by 7 and 12% for Phe and PAH mixed treatments respectively while an increase in shoot biomass yield by 7 and 2% was observed for Phe and B[*a*]P treatments respectively. As for *F. arundinacea* root biomass, 170, 86, and 45% yield increase was observed in the Phe, Flu, and B[*a*]P treatments respectively compared to the control. A root biomass decrease of 4% was seen in *F. arundinacea* root after the mixed PAH treatment. Post hoc test revealed that the root biomass for the Phe and Flu treatments was not different from each other but different from those of the mixed PAH and control without PAH (Fig. 1). The shoot/root dry weight ratios for *M. sativa* were 1.57, 1.30, 1.68, 2.63, and 2.33 in the control, Phe, Flu, B[*a*]P, and mixed PAH treatments respectively. The shoot/root dry weight ratios in the control, Phe, Flu, B[*a*]P, and mixed PAH treatments were 0.63, 0.19, 0.47,0.50, and 0.58 for *L. perenne* and 0.97, 0.33, 0.56, 0.68, and 0.89 for *F. arundinacea* respectively.

### PAH dissipation

The initial PAH concentrations in the single PAH treatments were phenanthrene 222 ± 40.6 mg kg^−1^, fluoranthene 104 ± 18.6 mg kg^−1^, and benzo[*a*]pyrene 2.08 ± 0.208 mg kg^−1^ and those for the mixed PAH treatment were phenanthrene 254 ± 42.2 mg kg^−1^, fluoranthene 153 ± 17.7 mg kg^−1^, and benzo[*a*]pyrene 2.65 ± 0.560 mg kg^−1^ (mean ± SE, number of replicate = 6). At end of the greenhouse experiment, there was a decrease in PAH concentration in the single and mixed PAH treatments of the planted soils and unplanted controls. The PAH loss was greater in planted soils compared to unplanted controls for benzo[*a*]pyrene while phenanthrene and fluoranthene dissipation in planted soils was slightly greater or equal to those of the unplanted controls. PAH loss in the treatments varied between compounds for *M. sativa*, *L. perenne*, and *F. arundinacea* after the phytoremediation experiment.

#### PAH dissipation in single PAH treatments

Phenanthrene and fluoranthene dissipation was comparable in the single treatments with *F. arundinacea* and *L. perenne* and exceeded those of the controls (both abiotic and unplanted controls). It was observed that those of *M. sativa* were statistically different to the abiotic control but similar to the unplanted control (Table 1). The Tukey test revealed that the residual concentration of phenanthrene across the plants was different from that of the abiotic control (*p* < 0.05). Abiotic processes and rhizodegradation contributed to 68% and 30–31% of the total phenanthrene dissipation respectively (Table 1). For fluoranthene dissipation, abiotic processes and rhizodegradation accounted for 41% and 52–58% of the overall loss respectively (Table 1). For the benzo[*a*]pyrene treatment, the outcome was different, and the residual B[*a*]P concentrations for *L. perenne* (1.92 ± 0.434 mg kg^−1^) and *F. arundinacea* (0.579 ± 0.123 mg kg^−1^) were significantly different from each other. Abiotic processes and rhizodegradation accounted 14% and − 6–58% of the total benzo[*a*]pyrene dissipation respectively.

**Table 1:** Phenanthrene, fluoranthene, and benzo[*a*]pyrene dissipation from single and mixed PAH treatments with *M. sativa*, *F. arundinacea*, and *L. perenne*. (Average values ± SE, *n* = 3). Different letters in each group indicate a significant difference at *p* < 0.05 according to Tukey’s HSD test. *Asterisked (negative) values represent percentage inhibition. Initial PAH concentration of single PAH treatments: phenanthrene (222 ± 40.6 mg kg^−1^), fluoranthene (104 ± 18.6 mg kg^−1^), benzo[*a*]pyrene (2.08 ± 0.208 mg kg^−1^). Initial PAH concentration of mixed PAH treatment: phenanthrene (254 ± 42.2 mg kg^−1^), fluoranthene (153 ± 17.7 mg kg^−1^), benzo[*a*]pyrene (2.65 ± 0.560 mg kg^−1^)

PAH treatment	PAHs	Plant/control	Mean residual concentration (mg kg^−1^)	Total PAH loss (%)	Rhizodegradation (% contribution by plants and microbes)
Single	Phe	*M. sativa*	3.62 ± 2.88a	98	30
		*L. perenne*	1.53 ± 0.0918ab	99	31
		*F. arundinacea*	1.99 ± 0.0885ab	99	31
		Abiotic control	70.7 ± 0.740b	68	
		Unplanted control	4.05 ± 2.01ab	98	
	Flu	*M. sativa*	7.51 ± 0.488a	93	52
		*L. perenne*	1.06 ± 0.146a	99	58
		*F. arundinacea*	0.830 ± 0.294a	99	59
		Abiotic control	61.7 ± 3.91b	41	
		Unplanted control	6.92 ± 1.69a	93	
	B[*a*]P	*M. sativa*	1.59 ± 0.132ab	24	10
		*L. perenne*	1.92 ± 0.434a	8	− 6*
		*F. arundinacea*	0.579 ± 0.123b	72	58
		Abiotic control	1.79 ± 0.190ab	14	
		Unplanted control	1.58 ± 0.320ab	24	
Mixed	Phe	*M. sativa*	36.2 ± 29.1a	86	19
		*L. perenne*	2.10 ± 0.260b	99	33
		*F. arundinacea*	1.74 ± 0.400b	99	33
		Abiotic control	85.4 ± 4.03a	66	
		Unplanted control	1.78 ± 0.350b	99	
	Flu	*M. sativa*	22.4 ± 5.10a	85	24
		*L. perenne*	1.87 ± 0.190b	98	38
		*F. arundinacea*	3.71 ± 1.54b	98	36
		Abiotic control	59.3 ± 3.18c	61	
		Unplanted control	8.54 ± 2.21b	94	
	B[*a*]P	*M. sativa*	1.66 ± 0.130a	37	− 11*
		*L. perenne*	2.15 ± 0.0600b	19	− 29.1*
		*F. arundinacea*	0.780 ± 0.01c	71	22.6
		Abiotic control	1.39 ± 0.100a	48	
		Unplanted control	1.33 ± 0.120a	50	

#### PAH dissipation in mixed PAH treatment

The PAH dissipation varied between compounds, phenanthrene (86–99%), fluoranthene (85–99%), and benzo[*a*]pyrene (37–71%). The mean residual concentration of phenanthrene was highest for the abiotic control with 85.4 ± 4.03 mg kg^−1^ and lowest for *F. arundinacea* with 1.74 ± 0.40 mg kg^−1^ as residual concentration. The residual concentration of phenanthrene and fluoranthene of the mixed PAH treatment with *M. sativa* differed significantly from those involving *F. arundinacea* and *L. perenne* (*p* < 0.01). B[*a*]P dissipation was greatest for *F. arundinacea* (1.87 mg kg^−1^; 71%) and lowest for *L. perenne* (0.5 mg kg^−1^; 19%) and was significantly different (*p* < 0.01) (Table 1).

## Discussion

Phytoremediation is an eco-friendly and sustainable remediation technology for contaminants including PAHs. However, there are conflicting findings on the phytoremediation outcome and few studies on mixed PAH contamination that may reflect real site scenarios. The present study assessed the impact of single and mixed PAH treatments on plant biomass yield and PAH dissipation during a greenhouse experiment. The contribution of abiotic processes and rhizodegradation to the overall PAH dissipation was also assessed. Contrary to our hypothesis, single and mixed PAH treatments either had stimulatory or inhibitory effects on plant biomass yields. Also, the impact of single and mixed PAH treatments on plant biomass yields was not different. B[*a*]P dissipation was enhanced in treatments with *F. arundinacea* but inhibited in those with *L. perenne* and *M. sativa. L. perenne* inhibited B[*a*]P dissipation to a greater extent than *M. sativa* in both treatments. Phe and Flu dissipation was inhibited in vegetated PAH treatments. Abiotic processes such as volatilization and soil adsorption were more important as dissipation mechanisms compared to rhizodegradation in both treatments.

The inhibitory effect of PAH contamination on biomass yields of *L. perenne* and *F. arundinacea* is attributed to single or synergistic effect of PAHs in the single and mixed PAH treatments respectively. However, an increase in plant biomass yields and shoot to root ratio especially for *M. sativa* is contrary to most reports on phytotoxic effect of PAH contamination. Cheema et al. (2010) reported a 35% decrease in *M. sativa* biomass in soils spiked with phenanthrene (200 mg kg^−1^) and pyrene (199 mg kg^−1^). The biomass yield increase of *F. arundinacea* in the Phe and Flu treatments compared to the mixed PAH and control may be attributed to the PAH concentration. Jeelani et al. (2017) reported a significant increase in biomass yield of *Acorus calamus* grown on soils spiked with phenanthrene (50–100 mg kg^−1^) and pyrene (25–50 mg kg^−1^). Increase in plant biomass yields in the B[*a*]P treatment agrees with the findings of Sun et al. (2011) that B[*a*]P concentration ≤ 10 mg kg^−1^ of enhanced biomass yield of *Tagetes patula*. Chigbo and Batty (2013) also reported an enhanced germination and shoot elongation of *L. perenne* with B[*a*]P concentration of 1–4 mg L^−1^. With the exception of the phenanthrene treatment, the increase in shoot to root ratio for *M. sativa* in PAH treatments is supported by Salehi-Lisar and Deljoo (2015) who reported an increase of up to 1.29 times for *M. sativa* in fluorene treatments (0–100 mg/kg). The decrease in shoot to root ratio for *L. perenne* and *F. arundinacea* relative to their control agrees with the findings of Salehi-Lisar and Deljoo (2015) for *Triticum aestivum.* Abiotic processes and microbial degradation had a greater contribution than rhizodegradation in vegetated treatments that inhibited phenanthrene and fluoranthene dissipation. Sun et al. (2010) reported a significant loss of phenanthrene (83.4%) and pyrene (57.2%) from freshly spiked sterile soil as a result of abiotic process especially volatilization. Inhibition of PAH dissipation in the presence of vegetation is contrary to many reports of an enhanced PAH dissipation during phytoremediation (Olson et al. 2007; Hall et al. 2011). In a recent report, phenanthrene and pyrene dissipation was not significantly affected by the presence of the plant, *Acorus calamus* when compared to the unplanted control (Jeelani et al. 2017). We observed that *L. perenne* inhibited B[*a*]P dissipation to a greater extent than *M. sativa* in both treatments.

PAH contamination has adverse effect on water and nutrient uptake by plants with impact on biomass yield (Reilley et al. 1996; Cheema et al. 2010; Oguntimehin et al. 2010). Phytotoxicity is dependent on plant stress tolerance and degradation capability of indigenous soil microbes (Kechavarzi et al. 2007). Increase in *M. sativa* biomass yield and shoot to root ratio indicates tolerance to PAH contamination as well as a stimulatory effect on plant growth (Hall et al. 2011). On other hand, a decrease in the shoot to root ratio for *L. perenne* and *F. arundinacea* indicates phytotoxic effects of the PAH treatments with impacts on plant development and senescence (Kechavarzi et al. 2007; Cheema et al. 2010). Although there are several reports on tolerance of *M. sativa* to PAH contamination, conflicting findings may be related to differences in soil properties and soil microbial community. Abiotic processes and microbial degradation are principal dissipation mechanisms for phenanthrene due its low molecular weight (178.23 g mol^−1^), vapor pressure (18 mPa), and solubility in water at 25 °C of 1.18 mg L^−1^ (Sun et al. 2010; Smith et al. 2011). However, volatilization is less likely to be a PAH dissipation mechanism for fluoranthene and benzo[*a*]pyrene with 3 or more rings and low vapor pressure. Also, high molecular weight PAHs adsorb to soil organic matter to facilitate formation of non-extractable bound residues thereby decreasing bioavailability (Kaimi et al. 2006; Hamdi et al. 2012). As such microbial degradation and rhizodegradation become relatively more important mechanisms for their dissipation in unplanted and planted soils respectively. Microbial degradation which involves mineralization, cometabolism, and non-specific radical oxidation depends on indigenous soil microorganisms, chemical properties of PAHs, soil properties, and environmental conditions (Smith et al. 2011; Toyama et al. 2011).

During phytoremediation, enhancement, or inhibition of PAH dissipation is determined by root exudate profile, catabolite repression, root morphology, and soil properties amongst others (Liste and Alexander 2000; Louvel et al. 2011; Jia et al. 2016). These factors also affect the extent of inhibition as observed with *L. perenne* compared to *M. sativa* for B[*a*]P dissipation. With respect to the effect of plants and root exudate on PAH dissipation, Guo et al. (2017) reported an enhanced PAH degradation at the early stage and then a decreasing effect as root exudates depleted. Differences in root exudate profile of *M. sativa* and *L. perenne* have impact on phytostimulation, gene expression, and plant-microbe interaction (Liste and Alexander 2000). Kamath et al. (2004) showed that *nah*G gene expression in *Pseudomonas fluorescens* HK44 was inhibited by some root extracts, sugars, and amino acids, hence affecting naphthalene degradation. Similarly, a decreased phenanthrene degradation activity of *Pseudomonas* spp. was observed following catabolite repression by root extracts such as pyruvate, glucose, and acetate (Louvel et al. 2011). Considering root morphology and density of *L. perenne*, aeration by the roots can create an oxidizing condition which suppresses some degradative reactions (Perelo 2010). Further, soil properties such as low pH and nutrient levels are critical for benzo[*a*]pyrene biodegradation. Unlike *L. perenne*, *M. sativa* may influence benzo[*a*]pyrene dissipation by altering soil pH. Nutrient depletions especially nitrogen due to competition between the plants and soil microbes have been shown to affect microbial degradation of PAHs (Fu et al. 2012). This may also explain the greater inhibitory effect on B[*a*]P dissipation by *L. perenne* compared to *M. sativa* with nitrogen fixing ability.

We found that the effect of single and mixed PAH contamination on plant biomass yield and PAH dissipation was not different. Our study findings support few reports on the stimulatory effect of PAH contamination on plant growth while most studies report phytotoxic effect of PAH contamination. A stimulatory effect in biomass yield without an enhancement of PAH dissipation may indicate impact of rhizosphere activities on PAH degradation. Enhancement or inhibition is dependent on a number of factors such as the plant species and soil microbial community which also affect relative contribution and importance of different dissipation mechanisms. Although the contribution of some dissipation mechanisms was assessed, presence of some microbes and extracellular enzymes in abiotic controls cannot be excluded as complete maintenance of abiotic control is difficult (Margesin et al. 2003; Kaimi et al. 2006). The contribution of specific abiotic processes such as volatilization and soil adsorption to the overall dissipation was not investigated in this study. Mechanisms behind inhibition or enhancement of PAH dissipation during the greenhouse experiment were not determined.

Our study findings raise questions on mechanisms that determine enhancement or inhibition of PAH dissipation as well as the efficiency and suitability of phytoremediation as a remedial strategy for PAH-contaminated sites which are usual complex and variable. Further studies are required to understand critical controls of PAH contamination, plant selection, and rhizosphere activities on soil microbial community structure, microbial gene expression, plant-microbe interaction, and remediation outcome. Ultimately, these factors amongst others may be responsible for the differences observed here and elsewhere in PAH dissipation between both single and mixed PAH in laboratory and field experiments (Dakora and Phillips 2002; Haichar et al. 2008; Wenzel 2009). These would provide insights to strategies to enhance phytoremediation such as rhizoengineering and rhizosphere metabolomics-driven approach.

## Conclusion

Mixed PAH contamination had a greater impact on plant biomass yield albeit non-significant and in most cases, there was a lower PAH dissipation in comparison to the single PAH treatment. Single or mixed PAH contamination can either inhibit or enhance plant growth. Enhancement of PAH dissipation in the presence of plants was only observed for single and mixed treatments with benzo[*a*]pyrene and *F. arundinacea*. Abiotic processes and microbial degradation were the most important PAH dissipation mechanisms. The PAH compound, presence of plants, and choice of plant species amongst other factors determine relative contribution of dissipation mechanisms and remediation outcome, enhancement, or inhibition of PAH dissipation.
